# Changes in the Blood-Brain Barrier Function Are Associated With Hippocampal Neuron Death in a Kainic Acid Mouse Model of Epilepsy

**DOI:** 10.3389/fneur.2018.00775

**Published:** 2018-09-12

**Authors:** Bing Chun Yan, Pei Xu, Manman Gao, Jie Wang, Dan Jiang, Xiaolu Zhu, Moo-Ho Won, Pei Qing Su

**Affiliations:** ^1^Jiangsu Key Laboratory of Integrated Traditional Chinese and Western Medicine for Prevention and Treatment of Senile Diseases, Department of Traditional Chinese and Western Medicine, Yangzhou University, Yangzhou, China; ^2^Department of Integrated Traditional Chinese and Western Medicine, Medical College, Yangzhou University, Yangzhou, China; ^3^Jiangsu Key Laboratory of Zoonosis, Jiangsu Co-innovation Center for Prevention and Control of Important Animal Infectious Diseases and Zoonoses, Yangzhou, China; ^4^Department of Neurology, Haian Hospital of Traditional Chinese Medicine Affiliated to Nanjing University of Chinese Medicine, Haian, China; ^5^Department of Neurobiology, School of Medicine, Kangwon National University, Chuncheon, South Korea

**Keywords:** autophagy related neuronal death, kainic acid, hippocampus CA3, tight junction proteins, PECAM-1

## Abstract

The kainic acid (KA)-induced epilepsy experimental model is widely used to study the mechanisms underlying this disorder. Recently, the blood-brain barrier (BBB) has become an innovative alternative treatment target for epilepsy patients. KA causes neuronal injury and BBB damage in this experimental epilepsy model but the mechanisms underlying epilepsy-related neuronal injury, autophagy, and BBB damage remain unclear. Therefore, the present study investigated the relationships among neuronal injury, the expressions of autophagy-related proteins, and changes in BBB-related proteins during the acute phase of epilepsy to further understand the mechanisms and pharmacotherapy of epilepsy. NeuN immunohistochemistry and Fluoro-Jade B (FJ-B) staining in the hippocampal CA3 region revealed that neuronal death induced by intraventricular injections of 10 μg/kg KA was greater than that induced by 3 μg/kg KA. In addition, there were transient increases in the levels of microtubule-associated protein light chain 3-II (LC3I/II) and Beclin-1, which are autophagy-related proteins involved in neuronal death, in this region 24 h after the administration of 10 μg/kg KA. There were also morphological changes in BBB-related cells such as astrocytes, endothelial cells (ECs), and tight junctions (TJs). More specifically, there was a significant increase in the activation of astrocytes 72 h after the administration of 10 μg/kg KA as well as continuous increases in the expressions of platelet endothelial cell adhesion molecule-1 (PECAM-1) and BBB-related TJ proteins (Zonula occludens-1 and Claudin-5) until 72 h after KA treatment. These results suggest that the overexpression of autophagy-related proteins and astrocytes and transient increases in the expressions of BBB-related TJ proteins may be closely related to autophagic neuronal injury. These findings provide a basis for the identification of novel therapeutic targets for patients with epilepsy.

## Introduction

Epilepsy is the sudden abnormal discharge of neurons that can lead to chronic occurrences of transient cerebral dysfunction (1). Approximately 70% of epileptic patients can control their symptoms with regular antiepileptic drug treatments but the remaining 30% do not respond to existing medications. This subset of patients is often diagnosed with temporal lobe epilepsy (2, 3), which is the most common type of epilepsy (4, 5). A mouse model of TLE induced by injections of kainic acid (KA) produces behavioral seizures and neuropathological lesions that are similar to those of TLE patients (6–8).

Autophagy is a type of programmed cell death that represents a highly conserved cellular mechanism of protein recycling (9) that can be caused by hypoxia, intracellular stress, and a variety of other mechanisms (10, 11). The biochemical actions of autophagosomes include the accumulation of microtubule-associated protein light chain 3-II (LC3-II), which are intracellular double-membraned vesicles that encompass organelles and the cytoplasm (12). Upon processing from its cytoplasmic form (LC3-I), LC-3 is inserted into the inner and outer limiting membranes of autophagosomes as LC3-II via covalent lipidation (13). It is thought that autophagy activation contributes to various neurodegenerative diseases (14).

The Blood-brain barrier (BBB) is a diffusion barrier, composed of brain microvascular endothelial cells (BMVEC), basement membrane system, astrocytes and pericytes, which plays an important role for maintaining homeostasis of the central nervous system (15, 16). BMVECs are an important morphological basis of the BBB (17) and the structural and functional integrity of these cells are essential for maintaining the function of the BBB via the production of inflammatory mediators, nutrition delivery, maintenance of ionic balance, stimulation of thrombosis, and clotting (18). In addition, the activity of platelet endothelial cell adhesion molecule-1 (PECAM-1), which is a member of the immunoglobulin family of cell adhesion molecules, is mediated by neutrophils and vascular endothelial cells (19). The adhesive properties of PECAM-1 enhance EC migration and restore barrier function in a wide variety of vascular permeability disorders (20). Tight junction (TJ) proteins are the top junctional complex and also play a critical role in the BBB. TJs represent a barrier between cells that is composed of transmembrane proteins (e.g., occludin), cytoplasmic attachment proteins (e.g., Zonula occludens-1), and cytoskeleton proteins that establish an impenetrable connection between BMVECs (21). Many studies have shown that changes in the expression of TJ proteins, variation in their distribution, and the abnormal functioning of their structures could destroy the integrity of the TJ barrier and lead to changes in BBB permeability (22). Adjacent ECs stabilize the EC cytoskeleton by tightening with TJ proteins and adhesion junction (AJ) proteins, a process that is regulated by astrocyte secretions and physical contact. In addition, astrocytes increase transendothelial electrical resistance and reduce permeability by interacting with EC receptors to increase connexin expression (23). The astrocyte–EC interface is crucial for BBB function by promoting the production and integrity of BBB components (24). Leaking from the BBB leads to cerebral edema, which is the leading cause of death from acute stroke (25), and BBB dysfunction has been observed in patients with multiple sclerosis (26), dementia, Alzheimer's disease (27, 28), and Parkinson's disease (29). However, few studies have investigated the expressions of BBB-related proteins in subjects with KA-induced epilepsy. Therefore, we assessed KA-induced chronological changes in the levels of PECAM-1 and TJ proteins that may be related to neuronal injury in the hippocampal CA3 region of mice.

## Materials and methods

### Experimental animals

Adult male ICR mice (postnatal 8 weeks) were approved by the comparative medicine center of Yangzhou University (Yangzhou, China) and used domestically for 1 week under standard conditions at 25 ± 2°C with a 12-h light/dark cycle and were allowed free access to water and standard chow. All experimental procedures were in accordance with the National Institutes of Health guidelines for the care and use of laboratory animals and the study protocol was approved based on ethical procedures and scientific care by the Yangzhou University-Institutional Animal Care and Use Committee (YIACUC-14-0015). All efforts were made to use the fewest animals possible and to minimize pain caused by the procedures.

### Treatment with kainic acid

A solution of KA and saline was administered into the unilateral ventricle (−1.0, −0.22, and −0.3 mm relative to bregma) of each mouse. Prior to surgery, all mice were anesthetized with 10% chloral hydrate (Aladdin, China) and placed into the stereotaxic apparatus. Subsequently, the animals were randomly divided into three groups (*n* = 14 each; 7 animals for histological analyses and 7 animals for biochemical analyses) as follows: normal saline group (control-group), 3 μg/kg kainic acid group (3 μg/kg KA-group), 10 μg/kg kainic acid group (10 μg/kg KA-group). Normal saline group (control-group), 3 μg/kg kainic acid group (3 μg/kg KA-group), 10 μg/kg kainic acid group were sacrificed after 3 days. Following the KA injection, the 10 μg/kg KA group was divided into four subgroups (*n* = 14 in each group; 7 animals used for histological analyses and 7 animals used for biochemical analyses) based on time: 6, 24, 48, and 72 h. After the injection, all animals were returned to their cages where seizure severity was assessed in 10 min intervals for up to 2 h using the modified Racine scale (30, 31): stage 0, normal behavior; stage 1, immobility; stage 2, forelimb and/or tail extension, rigid posture; stage 3, repetitive movements, head bobbing; stage 4, rearing and falling; stage 5, continuous rearing and falling; stage 6, tonic seizure or death. All the injected mice without stage 0 randomly were used in the later experiments.

### Tissue processing for histology and histological analysis

#### Tissue processing for histology

For histological analyses, all mice were anesthetized with 10% chloral hydrate (Aladdin) and then perfused with 0.1 M phosphate-buffered saline (PBS; pH 7.4) followed by 4% paraformaldehyde in 0.1 M phosphate-buffer (PB; pH 7.4). The brains were removed, post-fixed in the same fixative for 4 h, and cryoprotected overnight by infiltration with 30% sucrose. Afterwards, the frozen tissue was serially sectioned into 30 μm coronal sections using a cryostat (Leica; Wetzlar, Germany) and then collected into six-well plates containing PBS.

#### Histological analysis

KA primarily damages the hippocampus, and the hippocampal CA3 region is a vulnerable area subject to KA-induced epilepsy. In the present study, the KA procedure was conducted on frozen brain sections to identify the number of degenerative cells using Fluoro-Jade B (FJ-B) staining, which was performed as described previously (30, 32). Briefly, seven sections per animal were selected to quantitatively analyze every type of immunoreactivity. The brain tissue samples were sliced and dried, transferred to 0.06% potassium permanganate for 15 min, and then immersed in 0.0001% FJ-B solution containing 0.01% glacial acetic acid. Next, the brain tissue was soaked in xylene to dehydrate it and sealed with neutral gum. The immunoreactive intensity of the FJ-B staining was evaluated using optical density (OD), and an immunoreactive structure was obtained after an average greyscale conversion using the formula: OD = log (256/average gray level); the OD of the background was consistent with the area adjacent to the measurement area. After subtracting background density, the OD of the image file was calibrated to a percentage (relative OD [ROD]) using Adobe Photoshop Version 8.0 prior to analysis with NIH Image 1.59 software. To ensure objectivity using the same conditions, each test sample was measured under the same experimental conditions by two different observers.

### Immunohistochemistry

The immunohistochemical analyses were performed as described previously (33). The sections were sequentially treated with 0.3% hydrogen peroxide (H_2_*O*_2_) in PBS for 20 min and 5% normal serum in 0.01 M PBS for 30 min. The sections were next incubated with diluted rabbit anti-NeuN (1:1,000, Cell Signaling Technology), rabbit anti-GFAP (1:500, Abcam), rabbit anti-ZO-1(1:500, Thermo Fisher Scientific), rabbit anti-GLUT-1 (1:500, Abcam), goat anti-IBA-1 (1:500, Abcam), and mouse anti-PECAM-1 (1:500, Bio-Techne), overnight at 4°C. Subsequently, the sections were exposed to biotinylated goat anti-rabbit, goat anti-mouse or rabbit anti-goat IgG (1:250, Vector, Burlingame, CA). And they were visualized with 3, 3'-diaminobenzidine tetrahydrochloride in 0.01M PBS, and then mounted on Adhesion Microscope slides. Following the dehydration procedure, the sections were mounted in neutral balsam (Solarbio; Beijing, China). To establish the specificity of immunostaining, a negative control experiment was performed with pre-immune serum rather than primary antibody. Negative controls are no immunoreactivity in all structures. Digital images of the hippocampal subregions were captured with an image-analyzing system equipped with a computer-based microscope (Nikon Corporation; Tokyo, Japan). Structures immunoreactive for NeuN, GFAP, Iba-1, PECAM-1, and/or ZO-1 were increased × 20^*^10 for analysis and the figure of the NeuN immunohistochemistry was by enlarged × 4^*^10. The staining intensities of the structures immunoreactive for GFAP, Iba-1, PECAM-1, and ZO-1 were evaluated using OD, as described above (34).

### Western blot analysis

For Western blot analyses, the mice (*n* = 7 animals at each time) were sacrificed, the hippocampus was removed and dissected with a surgical blade, and the tissue samples were serially and transversely cut (400 μm) using a vibratome. The samples were preprocessed using a Total Protein Extraction Kit (KeyGEN; Nanjing, China), as previously described (35). Protein concentrations were determined using a Pierce BCA Protein Assay Kit (Thermo Scientific). Using 10% sodium dodecyl sulfate polyacrylamide gel electrophoresis (SDS-PAGE), equal amounts of protein (30 μg) were separated and transferred to nitrocellulose membranes (Millipore; Bedford, MA, USA), which were stripped and used for incubating the antibodies. To reduce background staining, the membranes were incubated with 5% bovine serum albumin (BSA) in tris-buffered saline (TBS) containing 0.1% Tween 20 (TBS-T) for 60 min followed by incubation with rabbit anti-ZO-1 (1:1,000, Thermo Fisher Scientific), Claudin 5 (1:1,000, Abcam), LC3A/B (1:1,000, Cell Signaling Technology), Beclin-1 (1:1,000, Cell Signaling Technology), and β-actin (1:3,000, Arigo) overnight at 4°C. Subsequently, the membranes were exposed to secondary goat anti-rabbit IgG (Santa Cruz, USA) for 2 h at room temperature and SuperSignal West Pico Chemiluminescent Substrate (Thermo Scientific; Rockford, USA) was used for protein detection. The results of the Western blot analyses were assessed and densitometric analyses for the quantification of the bands were performed using Quantity One Analysis Software (Bio-Rad). These data were used to count the ROD; the ROD ratio was calibrated as a percentage with the control group designated as 100%. Each blot represents at least three similar independent experiments.

### Statistical analysis

All data are presented as a mean ± standard error of the mean (SEM). Differences in the means among the groups were statistically analyzed with one-way analysis of variance (ANOVA) tests followed by Duncan's *post-hoc* tests. *P*-values < 0.05 were considered to indicate statistical significance.

## Results

### Racine scale

**Figure 1:**
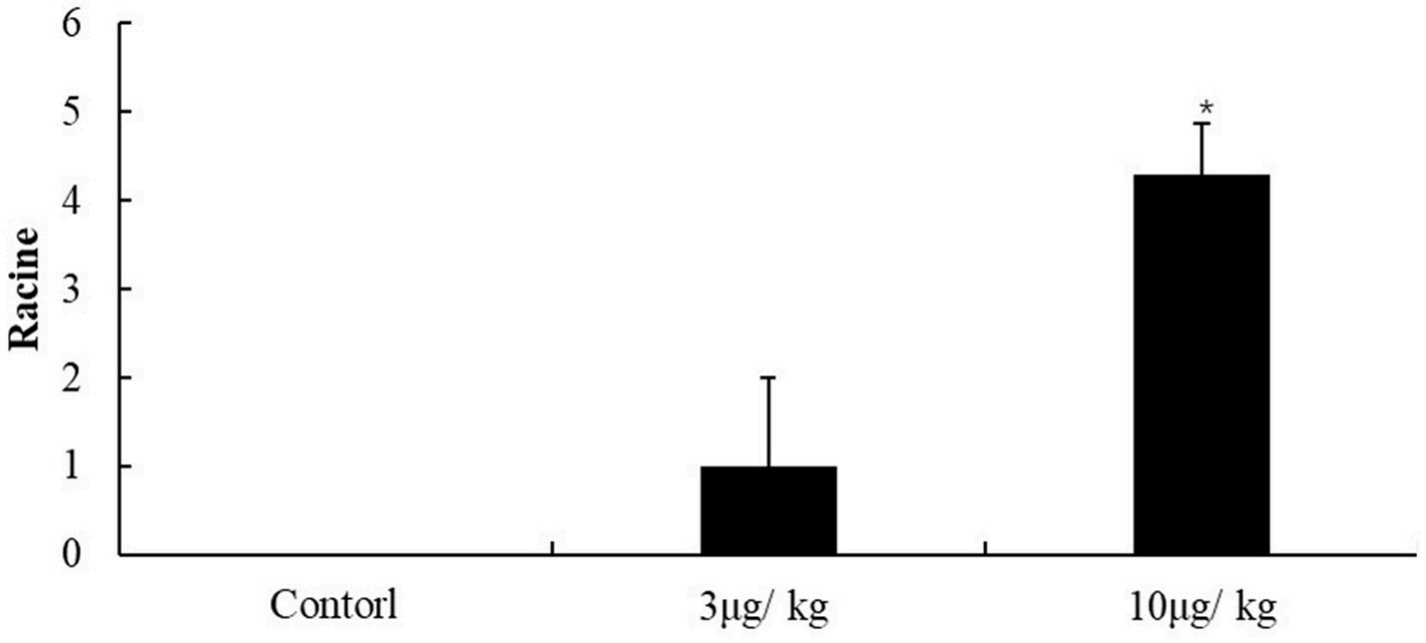
Racine scale. Data represent means ± SEM. ^*^P < 0.05 was compared with the control group. ANOVA tests followed by Duncan's *post-hoc* tests.

The seizure severity of mice was evaluated according to Racine's scale after KA injection (Figure 1). The scale in the control group were 0 that indicate no significantly seizure in mice, and in the 3 μg/kg KA-group, the scale were not significantly different with control group (about 1.5). The scores (about 4.5) in the 10 μg/kg KA-group were significantly higher than that in the control group (*p* < 0.05). These results indicate that the seizures in the 10 μg/kg KA group were more severe than those in the 3 μg/kg KA group and the control group.

### Neuronal survival in the all hippocampal subregions

**Figure 2:**
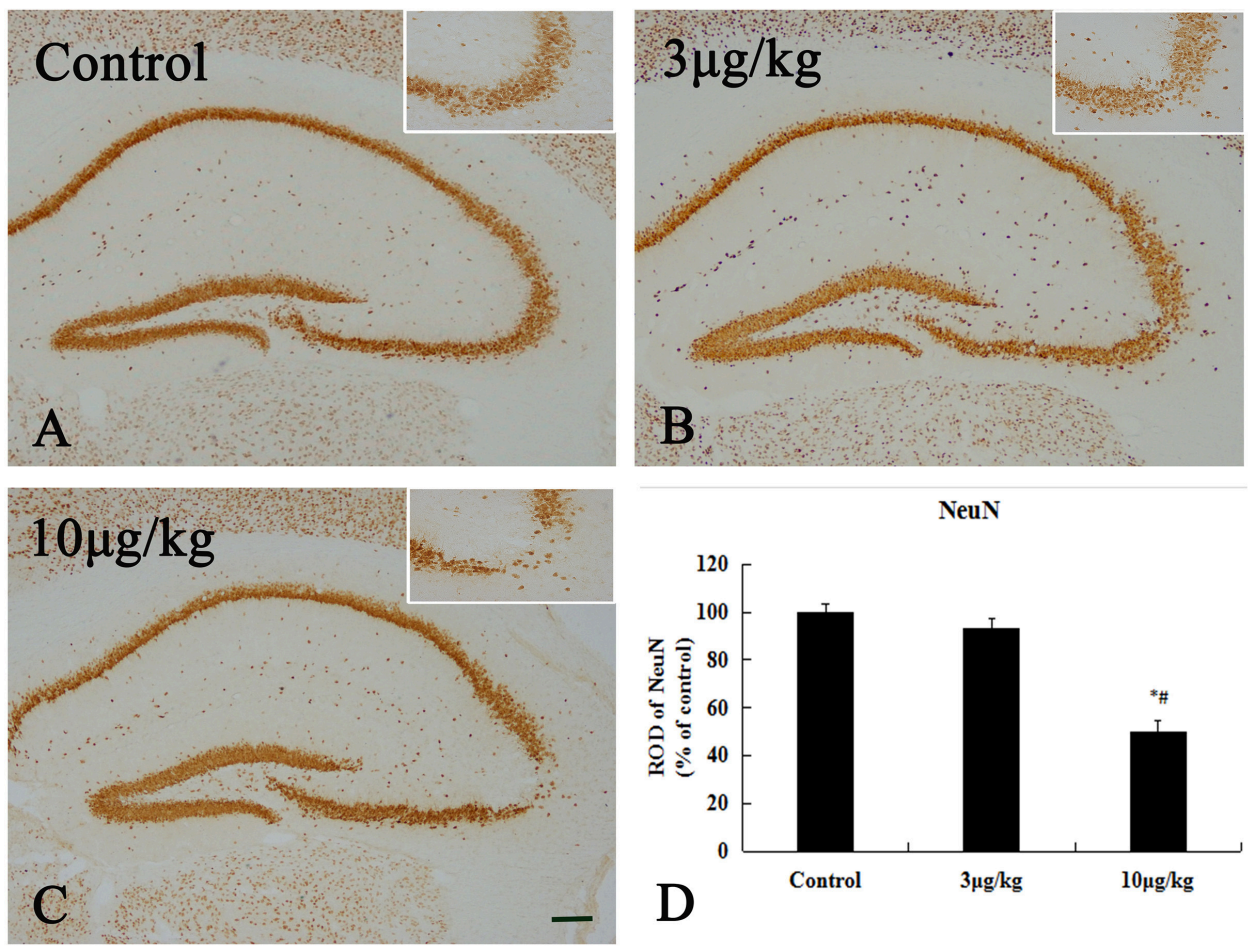
Decreased NeuN positive ^(+)^ cells induced by 10 μg/kg KA treatment in the CA3 area of the unilateral hippocampus. **(A–C)** Representative IHC images of NeuN^+^ cells in the unilateral hippocampus. **(D)** Quantification of NeuN^+^ cells for all treatment groups. Scale bars represent 250 μm. Data represent means ± SEM. ^*^P < 0.05, as compared with the control group. ^#^*P* < 0.05, as compared with the 3 μg/kg KA-group. ANOVA tests followed by Duncan's *post-hoc* tests.

Neuronal nuclei stained with NeuN were observed in the hippocampal region ipsilateral to the site of lesion (Figure 2 and Supplementary Figure 1). In the control group, many NeuN immunoreactive^(+)^ cells were observed in the all of regions in the hippocampus. The immunoreactivity of NeuN^+^ cells in the 3 μg/kg KA-group were almost equal with that in the control group (*p* > 0.05) (Figures 2A,B,D). After treatment for 72 h, the immunoreactivity and NeuN^+^ cells in the 10 μg/kg KA-group were significantly less than those in the control and 3 μg/kg KA-group, especially in the unilateral hippocampal CA3 region (*p* < 0.05) (Figures 2A–D). There were no other significant changes in the hippocampal neurons in any other region. Taken together, these findings suggest that the loss of neuronal cells caused by 10 μg/kg KA is more serious than the control group and 3 μg/kg KA-group.

### Neuronal death in the hippocampal CA3 region

**Figure 3:**
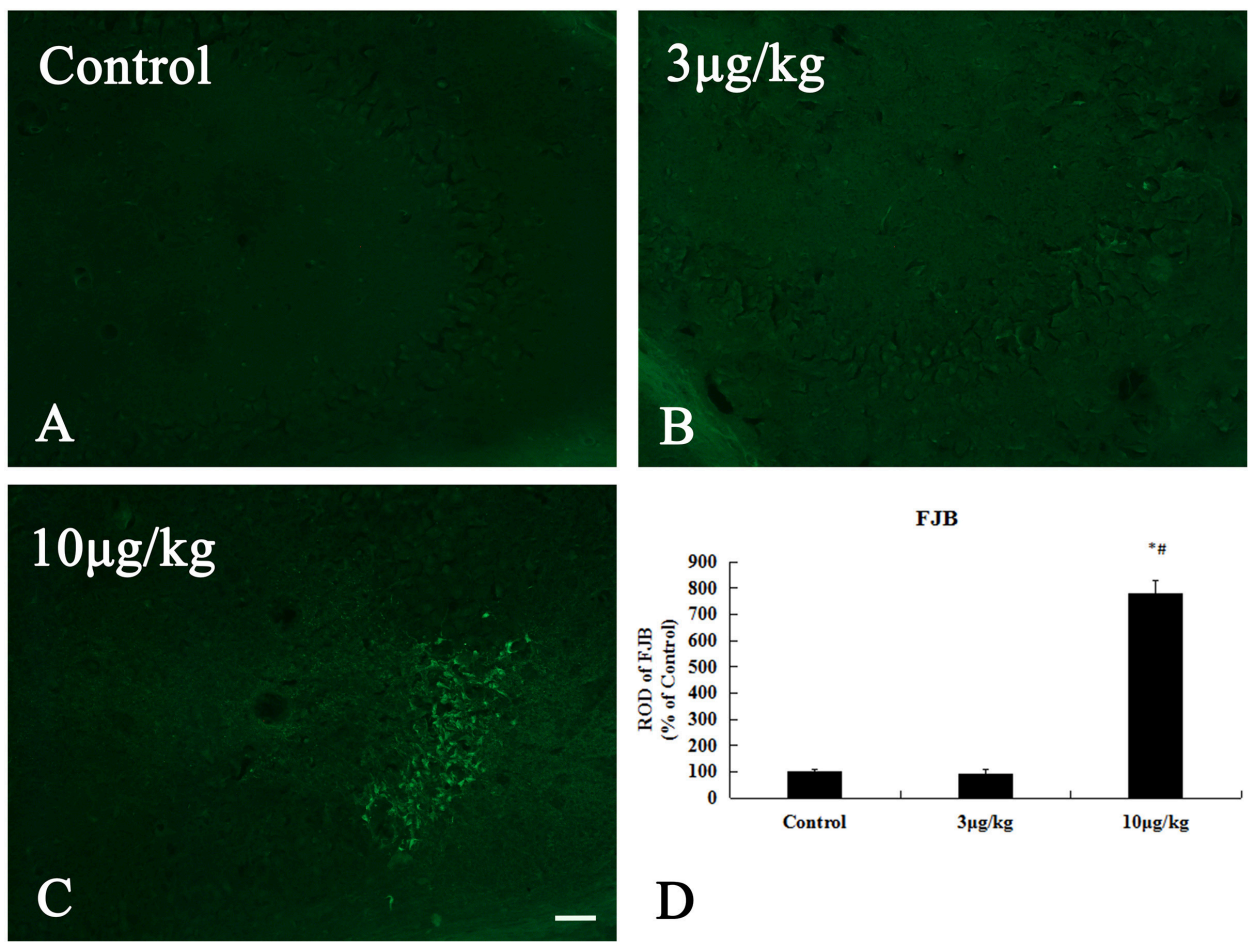
Increased neuronal death induced by 10 μg/kg KA treatment in the CA3 area of the unilateral hippocampus. **(A–C)** Representative IHC images of FJ-B staining in the CA3 area of the unilateral hippocampus. **(D)** Quantification of FJ-B ^(+)^ neurons for all treatment groups. Scale bars represent 50 μm. Data represent means ± SEM. ^*^P < 0.05, as compared with the control group. ^#^*P* < 0.05, as compared with the 3 μg/kg KA-group. ANOVA tests followed by Duncan's *post-hoc* tests.

In the control and 3 μg/kg KA-group, no FJ-B positive^(+)^ neurons were observed in all hippocampal subregion (Figures 3A,B). After 72 h treatment, in the 10 μg/kg KA-group, many FJ-B ^(+)^ neurons could be visualized in the unilateral hippocampal CA3 region (Figures 3C,D). The number of necrotic cells stained by FJB in the 10 μg/kg KA-group is more than other groups (*p* < 0.05). These data provide additional evidence that Neuronal death in the 10 μg/kg KA-group is more serious than the control group and 3 μg/kg KA-group.

### Expression of LC3 and beclin-1 protein levels in the hippocampus

**Figure 4:**
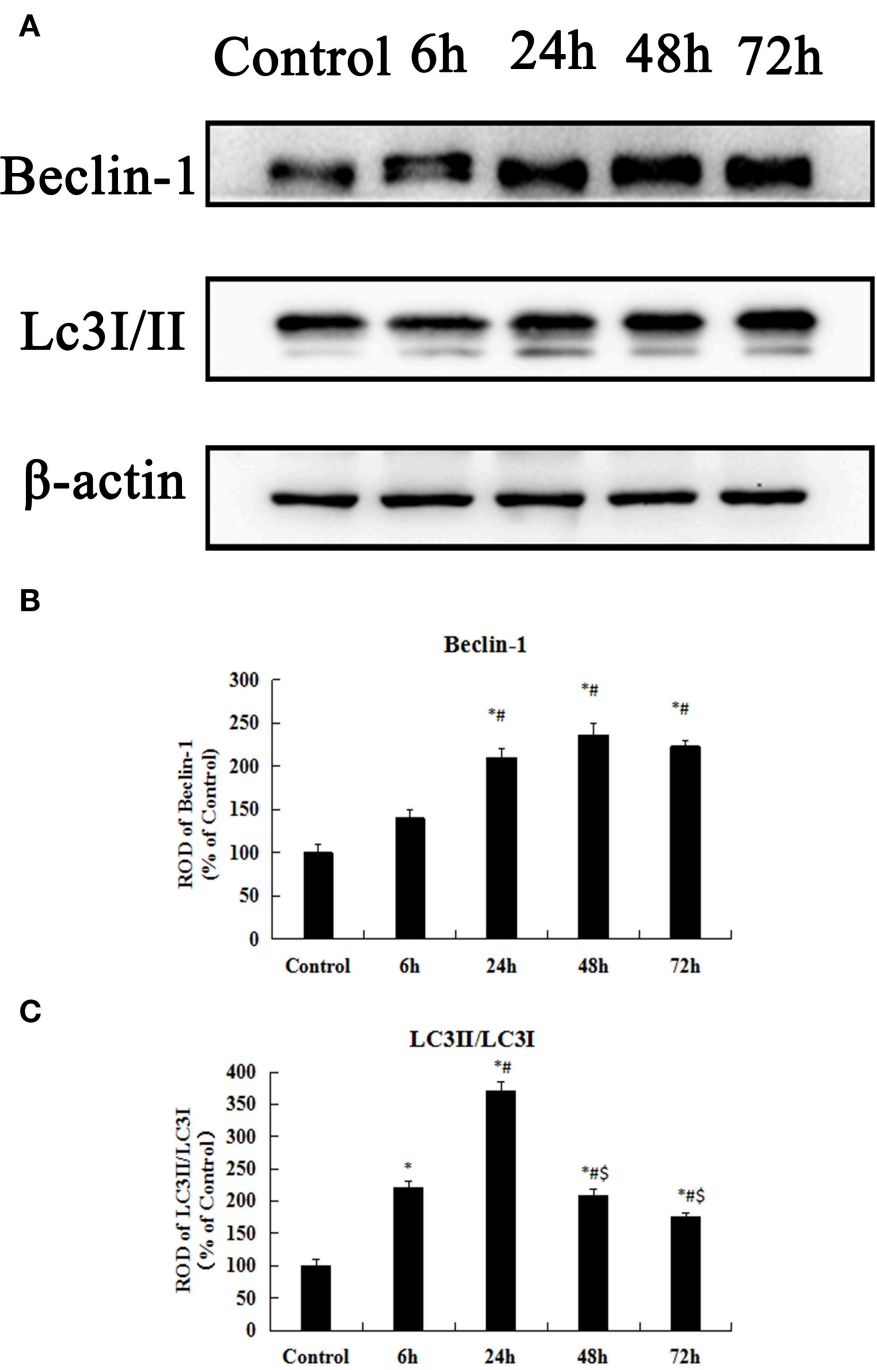
KA-induced neuronal injury in the hippocampus is associated with autophagic cell death. **(A)** Western blot analysis with antibodies against Beclin-1, LC3II/LC3I, and ACTIN. **(B–C)** Comparison of relative expression for Beclin-1 and LC3II/LC3I from the control group vs. the KA treatment groups. All results are expressed as means ± SEM (*n* = 7 per group). ^*^P < 0.05, as compared with the control group; ^#^*P* < 0.05, as compared with the 6 h KA-group; ^$^*P* < 0.05, as compared with the 24 h KA- group. ANOVA tests followed by Duncan's *post-hoc* tests.

To examine whether KA-induced neuronal injury in the hippocampus is likely associated with autophagic cell death, we test early chronological changes of Beclin-1 and LC3II levels. These results of western blot for the Beclin-1 and LC3II/LC3I expression levels in the hippocampus are shown in Figure 4. The expression of Beclin-1 was gradually increasing from control, 6 to 48 h after KA injection (*p* < 0.05) (Figures 4A,B). However, there was a small decrease at 72 h after the KA injection compared to 48 h after the KA injection. LC3II/LC3I levels in the hippocampus exhibited an increase at 6 h after the KA injection compared to the control group and its expression were peaked at 24 h after KA injection (*p* < 0.05); its expressions gradually decreased at 48 and 72 h after KA injection (*p* < 0.05) (Figures 4B,C). The present findings further indicate that KA-induced neuronal injury in the hippocampus was correlated with autophagic cell death.

### Microglia activation in the hippocampal CA3 region

**Figure 5:**
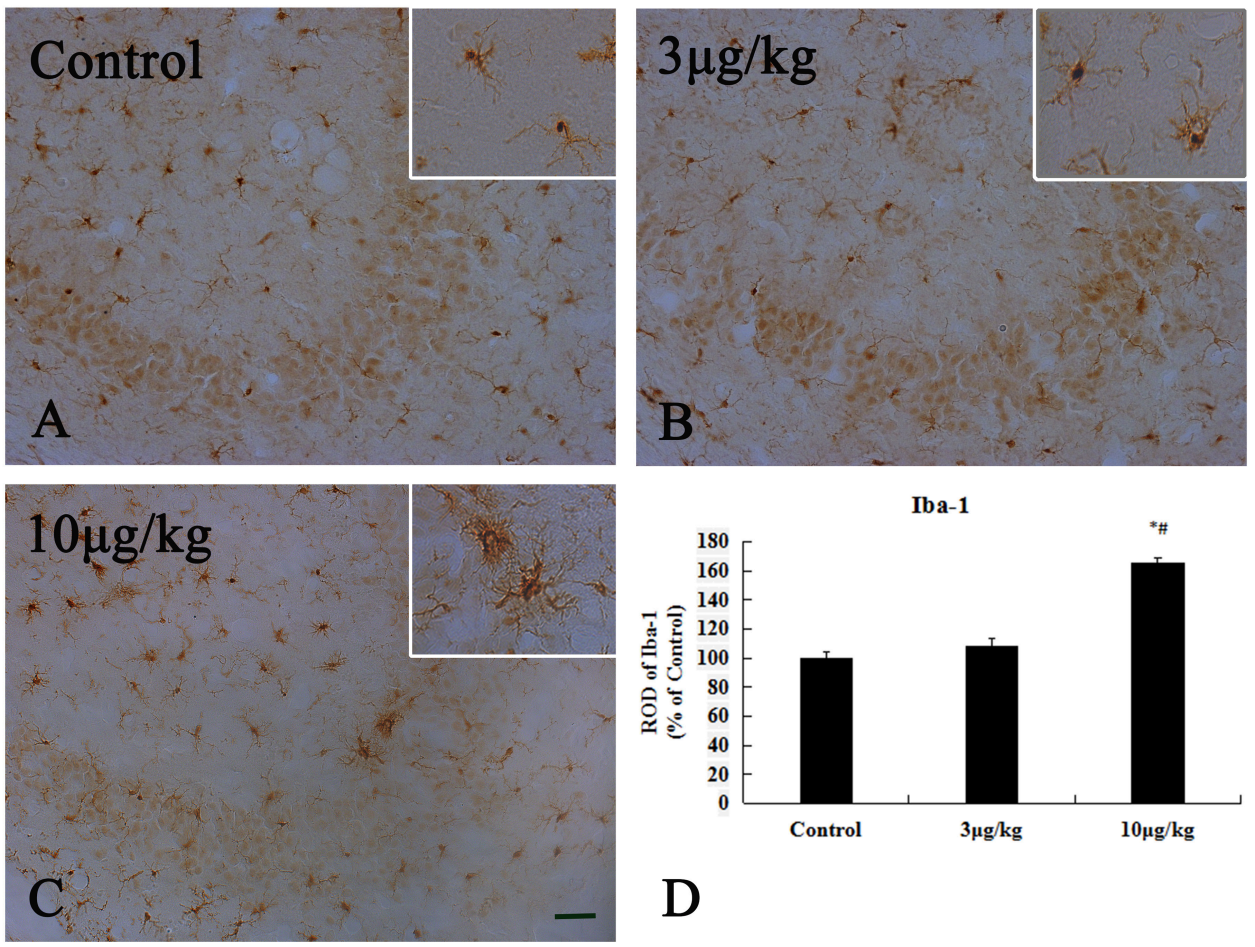
Activation of Iba-1^(+)^ microglia induced by 10 μg/kg KA treatment in the CA3 area of the unilateral hippocampus. **(A–C)** Representative IHC images of Iba-1^(+)^ cells in the CA3 area of the unilateral hippocampus. **(D)** Quantification of Iba-1^(+)^ cells for all treatment groups. Scale bars represent 50 μm. Data represent means ± SEM (*n* = 7 per group). ^*^P < 0.05, as compared with the control group. ^#^*P* < 0.05, as compared with the 3 μg/kg KA-group. ANOVA tests followed by Duncan's *post-hoc* tests.

Iba-1 is widely used as a specific marker for microglia in the CNS and many studies have reported microglial activation and subsequent upregulation of Iba-1 during CNS immune responses (36). In the present study, microglial activation was primarily detected in the hippocampal CA3 region (Figure 5 and Supplementary Figure 2). In the control group, Iba-1 immunoreactive microglia in the hippocampal CA3 region that exhibited characteristics of ramified or resting forms with fine processes in a web-like network. The degree of Iba-1 immunoreactivity and the number of activated microglia cells in the 10 μg/kg KA-group significantly increased in the hippocampal CA3 region compared to the control and 3 μg/kg KA-groups (*p* < 0.05) (Figures 5A,C). These data illustrate 10 μg/kg KA induced activated microglia in the hippocampal CA3 region.

### Astrocyte activation in the hippocampal CA3 region

**Figure 6:**
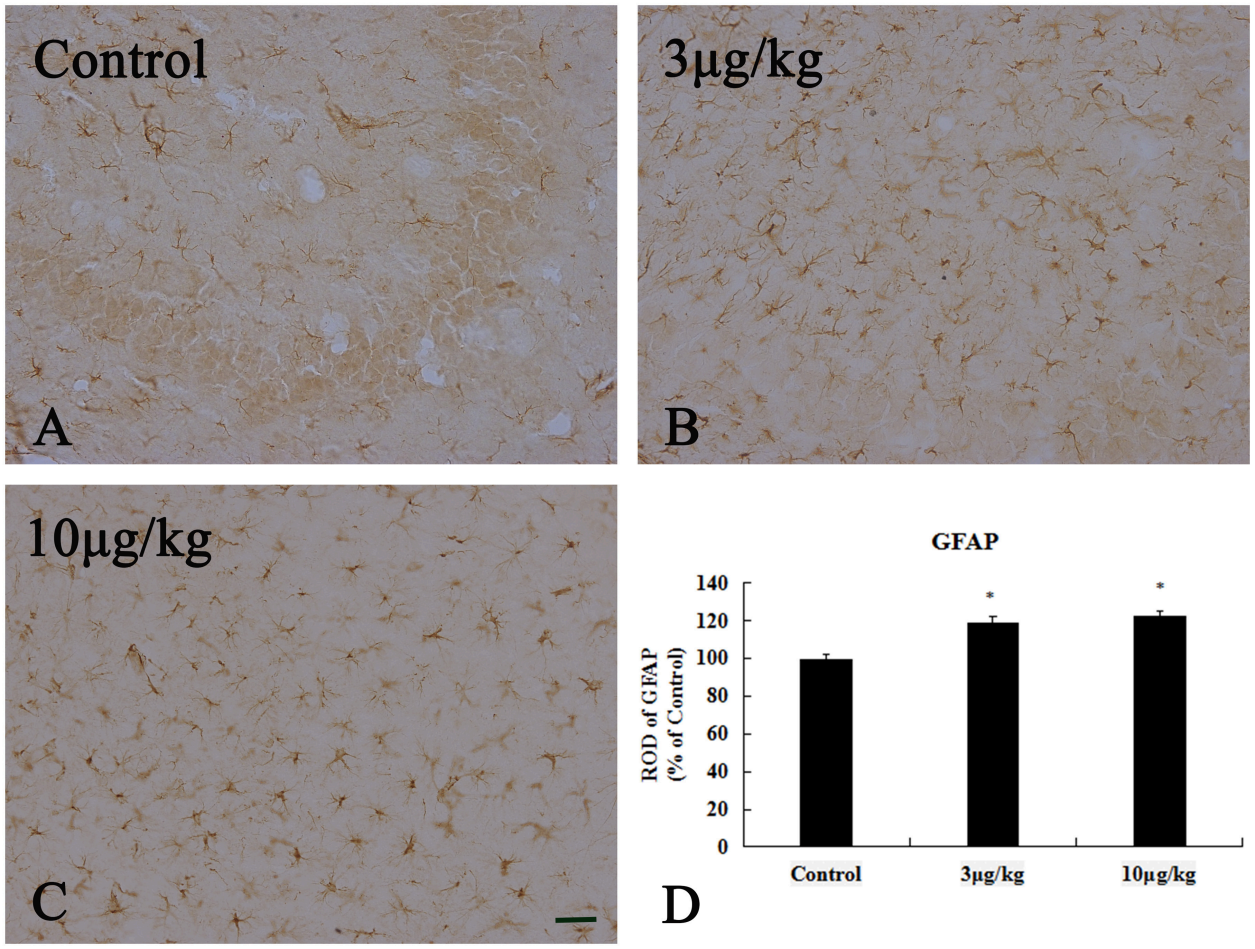
Activation of GFAP^+^ astrocyte induced by 10 μg/kg KA treatment in the CA3 area of the unilateral hippocampus. **(A–C)** Representative IHC images of GFAP^+^ cells in the CA3 area of the unilateral hippocampus. **(D)** Quantification of GFAP^+^ cells for all treatment groups. Scale bars represent 50 μm. Data represent means ± SEM (*n* = 7 per group). ^*^P < 0.05, as compared with the control group. ANOVA tests followed by Duncan's *post-hoc* tests.

The increased GFAP expression is widely used as a marker for astrogliosis. In the present study, we used the immunohistochemical analyses of GFAP to reveal differences in astrocyte activation between the control and KA-treated groups (Figure 6). In the control group, GFAP ^(+)^ astrocytes were distributed throughout the hippocampal CA3 region and showed a resting form with a small body and thin thread-like processes (Figure 6A). In the 3KA- and 10KA-groups, the activation and immunoreactivity of GFAP^+^ cells significantly increased compared to that in the control group (*p* < 0.05) (Figures 6B–D). We found that a certain dose of KA (10 μg/kg) injection could induce activated astrocytes in the hippocampal CA3 region.

### Expression of PECAM-1 and ZO-1 in the hippocampal CA3 region

**Figure 7:**
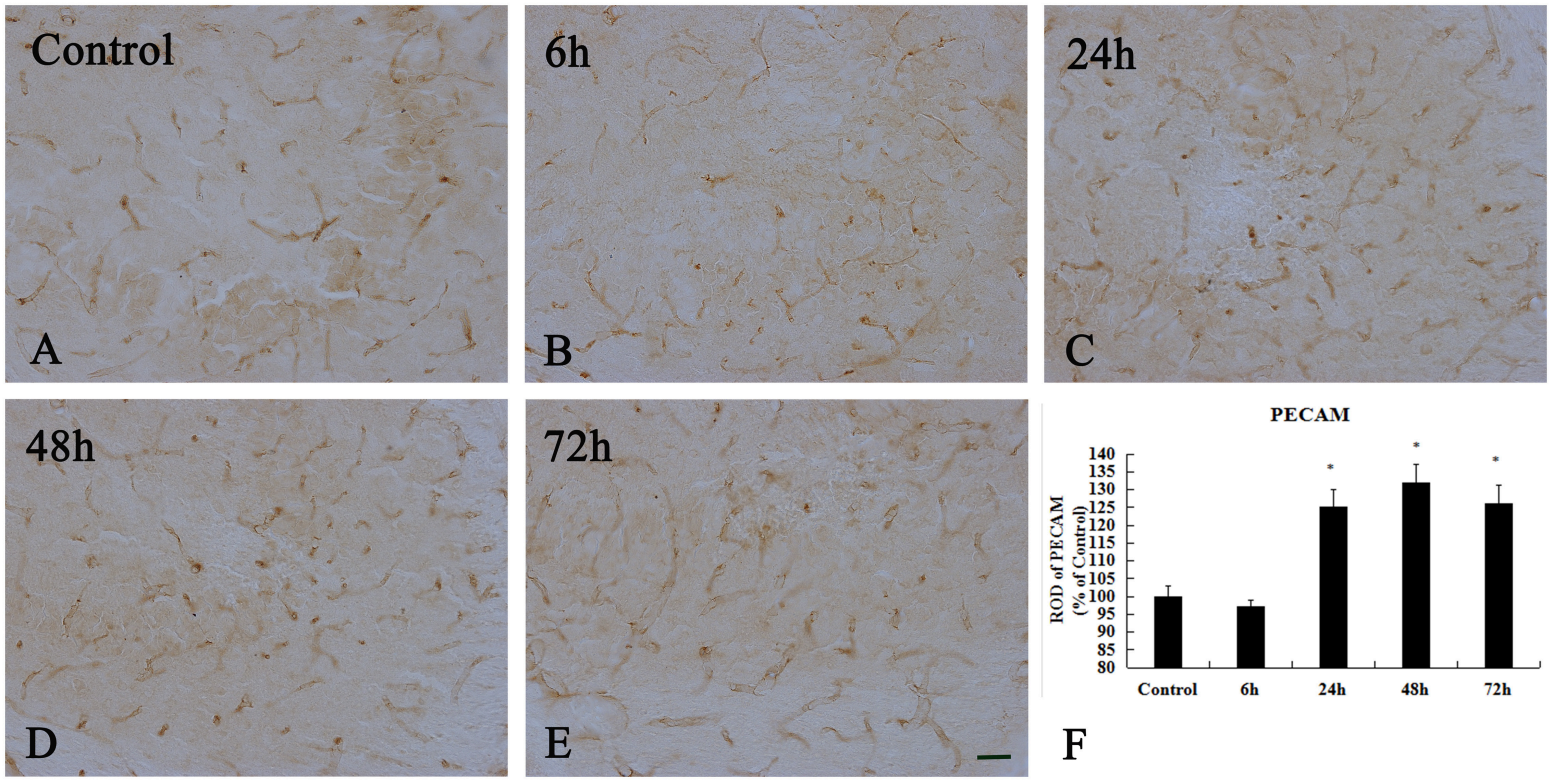
Enhanced immunoreactivity of PECAM^+^ cells induced by 10 μg/kg KA treatment in the CA3 area of the unilateral hippocampus. **(A–E)** Representative IHC images of PECAM^+^ cells in the CA3 area of the unilateral hippocampus. **(F)** Quantification of PECAM immunoreactivity for all treatment groups. Scale bars represent 50 μm. Data represent means ± SEM (*n* = 7 per group). ^*^P < 0.05, as compared with the control group. ANOVA tests followed by Duncan's *post-hoc* tests.

In the control group, PECAM-1 immunoreactivity was detected in the hippocampal CA3 region (Figure 7A). Our result showed that its expression at 6 h after KA injection was non-significant change in the hippocampal CA3 region compared to that in the control group (*p* > 0.05) (Figure 7B). PECAM-1 immunoreactivity was peaky increased in the hippocampal CA3 region at 24 and 48 h after KA injection(*p* < 0.05) (Figures 7C,D,F). At 72 h after KA injection, its immunoreactivity had somewhat decreased compared to that at 48 h, however, it remained higher than that of the control group (*p* < 0.05) (Figures 7E,F). We noted a significant increase in PECAM-1 immunoreactivity in the hippocampal CA3 region beginning at 24 h after injection of 10 μg/kg KA (*p* < 0.05).

**Figure 8:**
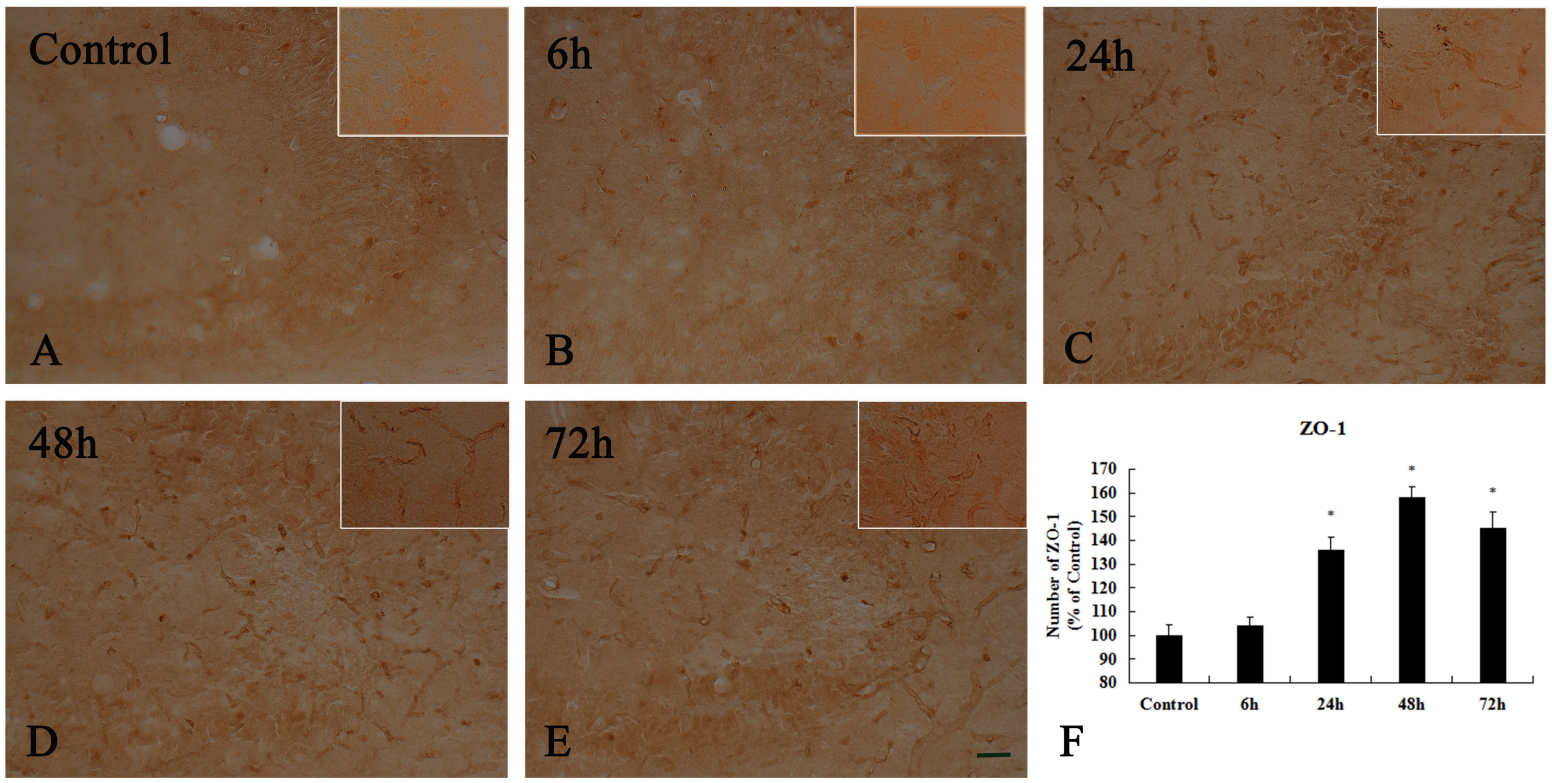
Enhanced activation of ZO-1^+^ cells induced by 10 μg/kg KA treatment in the CA3 area of the unilateral hippocampus. **(A–E)** Representative IHC images of ZO-1^+^ cells in the CA3 area of the unilateral hippocampus. **(F)** Quantification of ZO-1 immunoreactivity for all treatment groups. Scale bars represent 50 μm. Data represent means ± SEM (*n* = 7 per group). ^*^P < 0.05, as compared with the control group. ANOVA tests followed by Duncan's *post-hoc* tests.

To examine whether TJ-associated proteins, such as ZO-1, are associated with neuronal injury in the hippocampal CA3 region, we detected the level of ZO-1 by immunohistochemistry (Figure 8 and Supplementary Figure 3). In the control group and 6 h after KA injection, weak ZO-1 immunoreactivity was detected in the hippocampal CA3 region (*p* < 0.05) (Figures 8A,B). At 24, 48, and 72 h after injection, their immunoreactivities were significantly higher than those in the control group in the hippocampal CA3 region, however, there are not exhibit differences in ZO-1 immunoreactivity in the hippocampal CA3 region among them (*p* < 0.05) (Figures 8C–F).

### ZO-1 and claudin-5 protein levels expressed in the hippocampus

**Figure 9:**
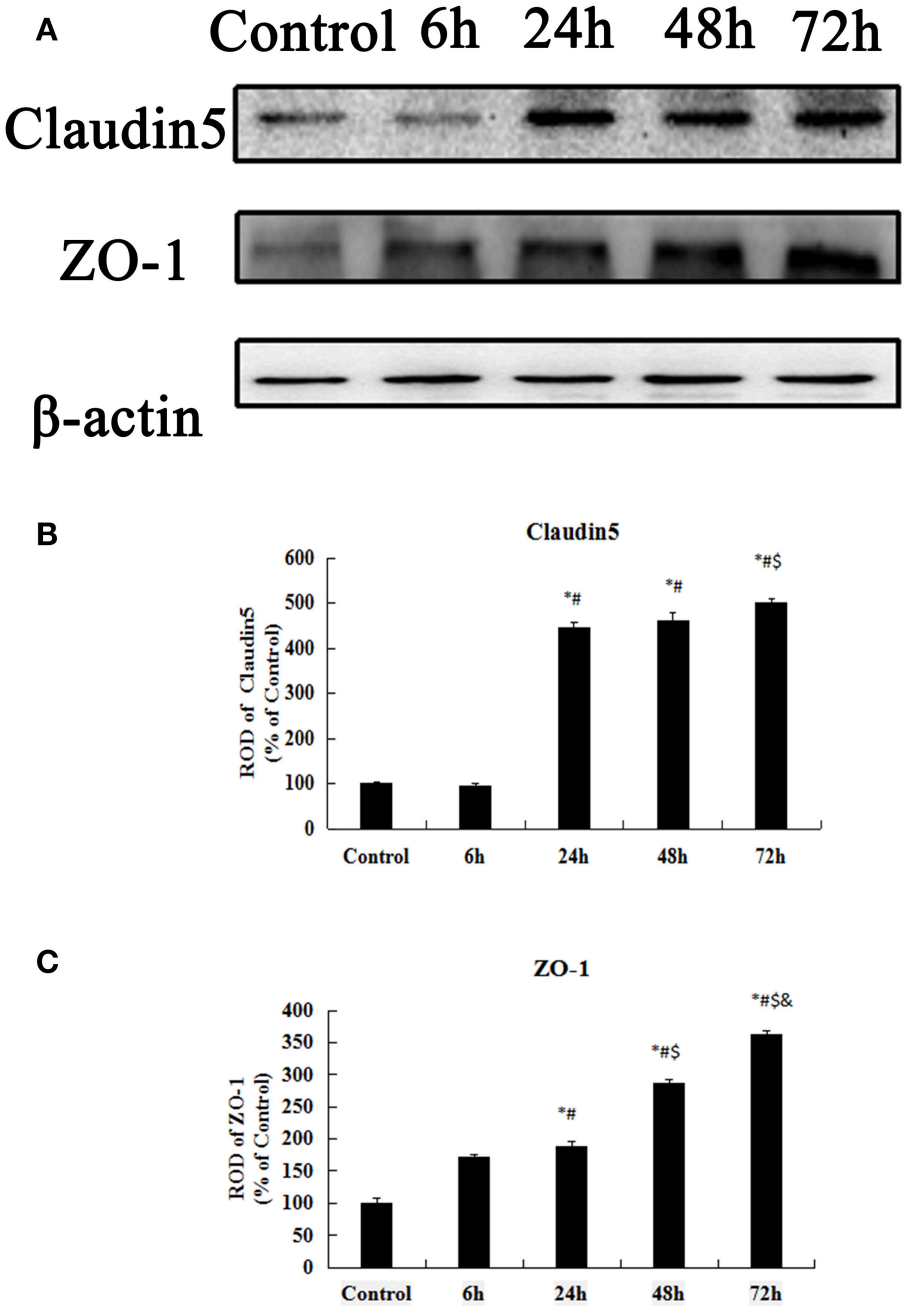
Western blot analysis for Claudin-5, ZO-1. **(A)** Western blot analysis for Claudin-5, ZO-1. **(B–C)** ROD, a percentage of the immunoblot band. All results are expressed as means ± SEM (*n* = 7 per group). ^*^P < 0.05, as compared with the control group; ^#^*P* < 0.05, as compared with the 6 h KA-group; ^$^*P* < 0.05, as compared with the 24 h KA- group; ^&^*P* < 0.05, as compared with the 48 h KA- group. ANOVA tests followed by Duncan's *post-hoc* tests.

The Western blot results of the ZO-1 and Claudin-5 levels in the control group and KA-injected groups are presented in Figure 9. Claudin-5 is a 23 kilodalton four-transmembrane protein known to form TJs between ECs at the BBB (37). There was no obvious difference in the expression level of Claudin-5 between the control group and the other group at 6 h after injection (*p* > 0.05) (Figures 9A,B). However, the expressions of Claudin-5 at 24, 48, and 72 h after KA injection were significantly higher than that in the control-group (*p* < 0.05) (Figures 9A,B).

ZO-1 links prejunctional F-actin and other cytoskeletal components that are highly associated with cytoskeleton and cell barrier function (38). ZO-1 protein levels in the hippocampus gradually increased from 6 h to 72 after injection (*p* < 0.05) (Figures 9A,C). Based on the above data, we found that the early transient increases in the expressions ZO-1, and Claudin-5 may be closely related to neuronal injury.

## Discussion

In the present study, neuronal death in the hippocampal CA3 region in a KA-induced epilepsy model was consistent with the results of previous studies (39). However, few reports have focused on the relationship between BBB injury and autophagy-related neuronal death. Therefore, changes in autophagy-related proteins, BBB-associated TJ proteins, astrocytes, and microglia were investigated in the acute phase of epilepsy because they may be closely related to the mechanisms underlying neuronal death.

### Autophagic cell death in KA-induced epilepsy model

The KA-induced epilepsy model has been widely used to investigate the mechanisms associated with epilepsy (40, 41). Some studies have shown that the neuronal injury caused by KA injections in the acute phase are the most serious (42) although autophagy has been induced in a variety of experimental epilepsy models, including KA- and pilocarpine-induced seizure models (43). Under normal conditions, autophagy is a salient cellular process that can either promote cell survival or cell death and is in dynamic equilibrium. Under pathological conditions, autophagosomes fuse with lysosomes to induce autophagic degradation, as in epilepsy, which can result in cell death via excessive autophagy. We observed significant chronological changes in neuronal death in the hippocampal CA3 region following an intracerebroventricular injection of 10 μg/kg KA as evidenced by NeuN immunohistochemistry and FJ-B staining. We also investigated whether neuronal death induced by KA injections would be associated with autophagic cell death. Beclin-1 and LC3II are commonly used markers of autophagy (44), and in the present study, the protein levels of Beclin-1 and LC3II/LC3I in the hippocampus gradually increased in a chronological manner until 48 h after the KA injection. When cells undergo autophagy, LC3I is converted into LC3II, which in turn is present on autophagosomal precursors and the autophagosomal membrane; thus, it is a specific marker of autophagosomes (13). Beclin-1 is an important regulatory gene for autophagy and has been extensively studied. This protein plays a role in the core complex with class III PI3K (45) and p150 (46) as a promoter of autophagy (47), which is affected in a variety of common neurodegenerative diseases and hereditary peripheral nervous system diseases (48). Overactivation of autophagy leads to cell death via mitochondrial degeneration and chromatin breakage (49) and KA-induced neuronal injury may be related to the activation of autophagy in the mouse hippocampus (50). The present findings further indicate that KA-induced neuronal injury in the hippocampus is likely associated with early chronological changes in Beclin-1 and LC3II levels, which are correlated with autophagic cell death.

### The glial cell activation in KA-induced epilepsy model

Many studies have demonstrated that BBB leakage in experimental epileptic mice is coincident with morphological changes in BBB-related cells, including astrocytes and ECs (51). Furthermore, BMVEC injuries induced by diseases of the CNS, including cerebral ischemia and epilepsy, occur during the initial phase of BBB disruption and result in a poor prognosis for patients. In both the pilocarpine- and KA-induced epilepsy models, BBB damage is observed in all regions of the hippocampus and brain (52). In addition, BBB damage is closely related to the extent and duration of seizures and may promote the occurrence of epilepsy (53). Serum levels of albumin are significantly elevated after BBB damage and can induce epilepsy by activating inflammatory cytokines, decreasing the epilepsy threshold, and causing gap junction coupling (54, 55). We found that a certain dose of KA (10 μg/kg) activated astrocytes and microglia in the hippocampal CA3 region. Previous studies have shown that KA-induced epilepsy models are characterized by the activation of astrocytes and microglia (56) and that the proliferation and activation of glial cells in hippocampus are closely related to epilepsy induced-neuronal death (57). Astrocytes are crucial for maintaining cell architecture and homeostasis under normal brain conditions. In fact, some studies have proposed that “reactive astrocytosis” involves morphological changes and functional changes in astrocytes in response to brain injury (58, 59). Astrocytes are an important component of the BBB and reactive astrogliosis is highly associated with BBB failure (60), which is a pathophysiological process that results from neuronal injury during epilepsy (61, 62). One possible cause of BBB leakage in experimental epilepsy models may be related to morphological changes in astrocytes that can damage the functional BBB structure (55, 63). Excessive microglial activation may also contribute to the pathophysiological process of epilepsy (64, 65) because pathophysiological changes in the brain that are induced by activated microglia, such as inflammation, can compromise barrier function and kill neurons in the BBB (66). Thus, it is likely that the astrocytic and microglial activations observed in the present KA-induced experimental epilepsy model were related to neuronal death and BBB failure.

### The increased PECAM-1 expression in the KA induced the TLE model

PECAM-1, which plays a vital role in the migration of leukocytes through ECs, is a common marker of microvascular ECs (67). We noted a significant increase in PECAM-1 immunoreactivity in the hippocampal CA3 region beginning at 24 h after the injection of 10 μg/kg KA. Significant increases in PECAM-1 expression have been observed in a spinal cord ischemia model, focal cerebral ischemia model, and other peripheral ischemia models and may be due to neutrophil migration (68). PECAM-1 plays an important role in maintaining the integrity of the EC connection and modulating its connection stability (20). PECAM-1 inhibits the activation of circulating platelets and supports the integrity of EC-cell junctions, which protects the vascular bed against some types of stimulation, and also appears to play a significant role in these interrelated processes (20, 69). Furthermore, PECAM-1 endows the vascular endothelium with the ability to maintain vascular integrity (70). Under pathological conditions, early and transient increases in PECAM-1 may be associated with the response to stimuli such as ischemia and inflammation (71). Similarly, the transient endogenous increase in antioxidants, including superoxide dismutase (SOD), after ischemia reperfusion protects against oxygen free radicals induced by cerebral ischemia (72). In TLE models, there is an overexpression of vascular endothelial factor (VEGF) and early BBB failure during seizures, which is followed by gradual increases in angiogenesis that may be involved in TLE-induced neuronal death (73). Thus, it is possible to speculate that the increased early PECAM-1 expression observed in the hippocampal CA3 region after the KA injection in the present study was related to the epilepsy-induced neuronal death.

### The transient increase of tight junction-associated proteins in KA-induced epilepsy model

TJs are composed of claudins and TJ-associated marvel proteins (TAMPs), such as occludin and tricellulin, that are present to varying degrees in different EC beds, particularly those that require the tight regulation of vascular permeability, including the BBB (74). We observed significant increases in the immunoreactivity of ZO-1 and Claudin-5 in the hippocampal CA3 region at 24 h after the 10 μg/kg KA injection. The ZO-1 and Claudin-5 proteins are indispensable components of the BBB system (75, 76). In addition, the expression of ZO-1 maintains normal functioning in TJs and improves paracellular transport between ECs and endothelial monolayers (77, 78). Dysfunction in TJ-associated proteins occurs in a number of CNS diseases. For example, neurodegenerative diseases such as stroke and Alzheimer's disease are closely related to changes in TJ-related proteins (79–81), and experimental studies have shown that the expressions of ZO-1 and Claudin-5 exhibit long-term decreases during seizures (73, 82). Our results indicate that transient early increases in the expressions of TJ-associated proteins, such as ZO-1 and Claudin-5, and changes in PECAM-1 expression may be associated with neuronal injury in the hippocampal CA3 region following injections of KA.

**Figure 10:**
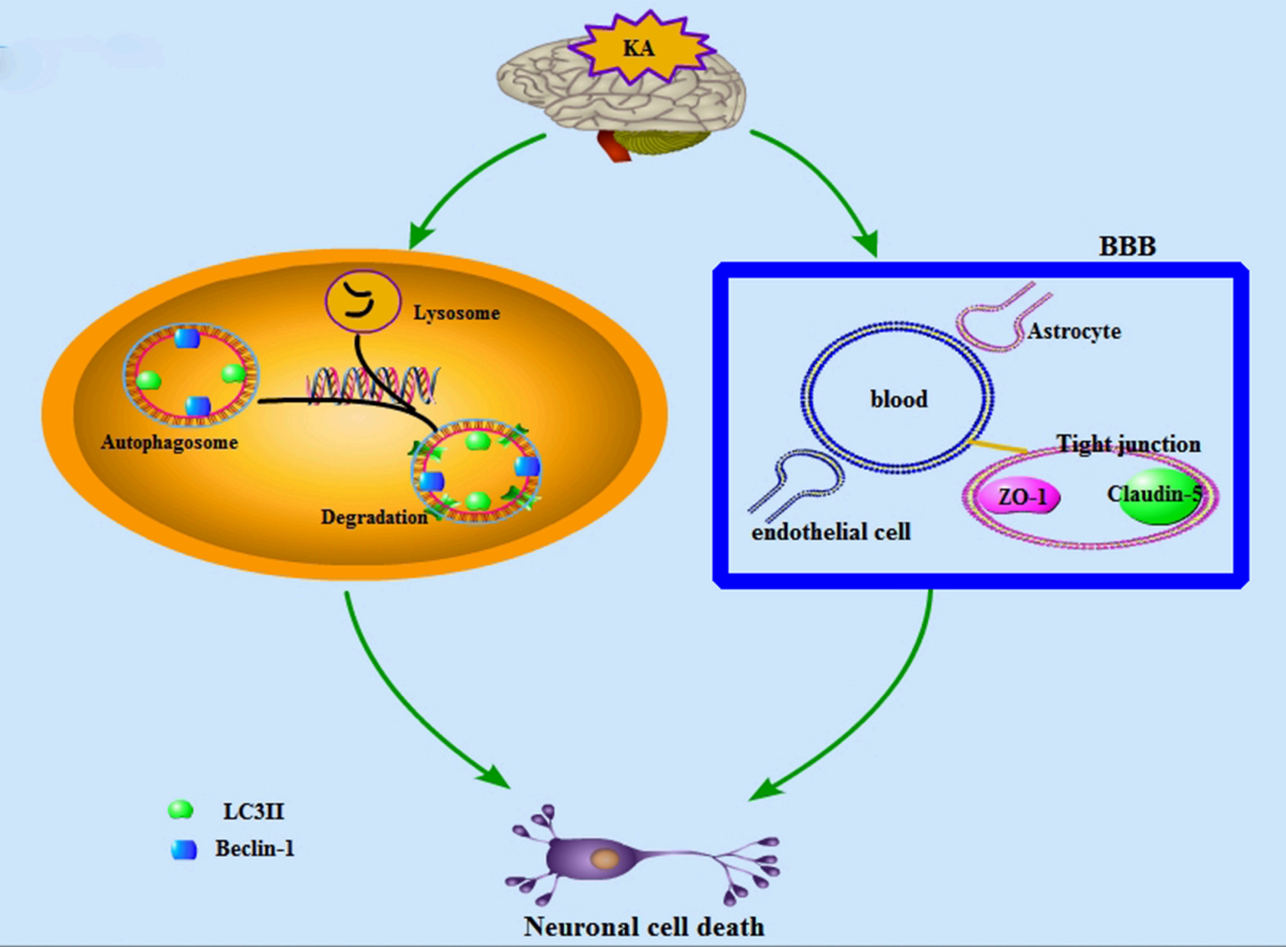
A schematic depiction of the KA-induced neuronal cell death which is associated with BBB damage and neuronal autophagic injury.

Our findings indicate that KA-induced neuronal death in the hippocampal CA3 region is related to autophagy. In addition, the overexpression of astrocyte activation and early transient increases in the expressions PECAM-1, ZO-1, and Claudin-5 may be closely related to neuronal injury (Figure 10). However, we only assessed changes in autophagy, the BBB, and neuronal injury during the acute phase of epilepsy. Further studies are necessary to verify the roles of autophagy and BBB damage in neuronal cell death using drugs. These results will provide novel therapeutic targets for the treatment of epilepsy.

## Author contributions

BY and PX participated in all aspects of the experimental design, analysis, and writing including obtaining, analyzing, and interpreting data and making significant contributions to the writing of the manuscript. MG carried out the experiments and computational modeling. JW, DJ, XZ, M-HW, and PS contributed to data analysis.

### Conflict of interest statement

The authors declare that the research was conducted in the absence of any commercial or financial relationships that could be construed as a potential conflict of interest.
