# Supplementary figures and images for: Changes in the Blood-Brain Barrier Function Are Associated With Hippocampal Neuron Death in a Kainic Acid Mouse Model of Epilepsy
Source: Front Neurol. 2018 Sep 12;9:775. doi: 10.3389/fneur.2018.00775 (PMC6143688; doi:10.3389/fneur.2018.00775)

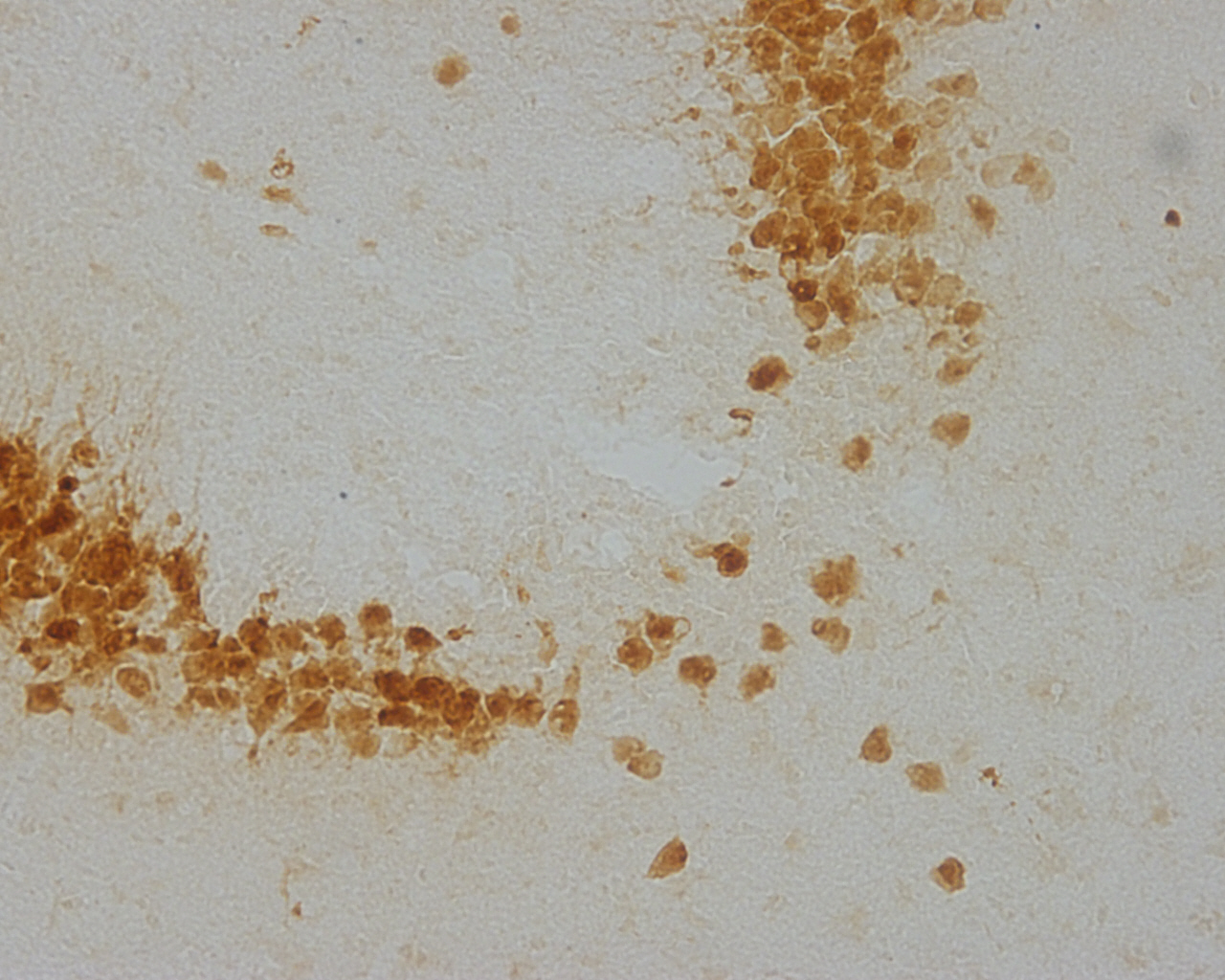

Supplement: Supplementary file 1 [file Image_1.TIF]

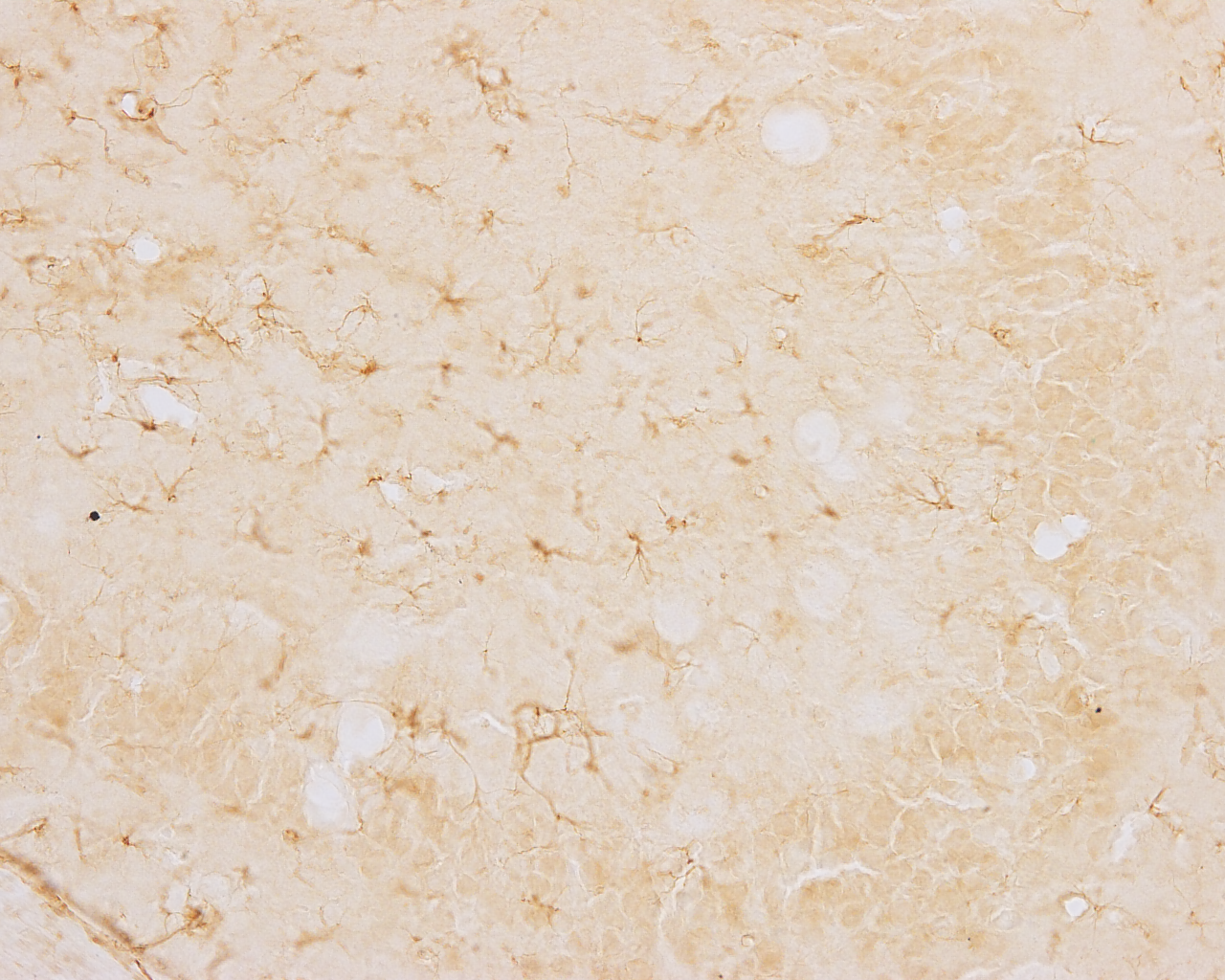

Supplement: Supplementary file 2 [file Image_2.TIF]

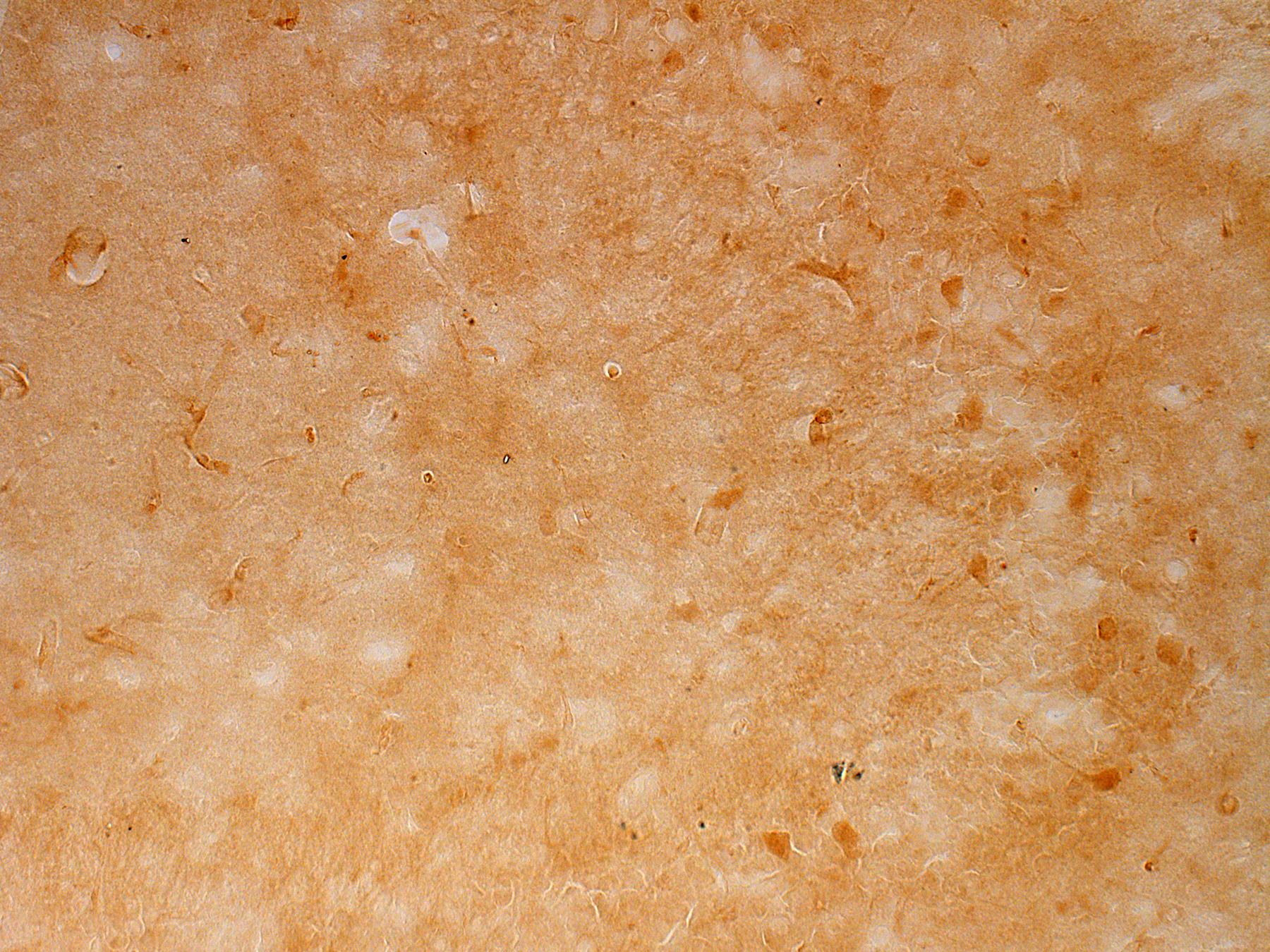

Supplement: Supplementary file 3 [file Image_3.TIF]
